# Supplementary material for: An adaptive threshold neuron for recurrent spiking neural networks with nanodevice hardware implementation
Source: Nat Commun. 2021 Jul 9;12:4234. doi: 10.1038/s41467-021-24427-8 (PMC8270926; doi:10.1038/s41467-021-24427-8)
Supplement: Supplementary file 1 — Supplementary Information File [file 41467_2021_24427_MOESM1_ESM.pdf]

Supplementary Information

A Novel Adaptive Threshold Neuron for Recurrent Spiking Neural Networks with Nanodevice Hardware Implementation

Ahmed Shaban[1] , Sai Sukruth Bezugam[1] , and Manan Suri[1]*

[1]Indian Institute of Technology, Electrical Engineering, Delhi, 110016, India *manansuri@ee.iitd.ac.in

*** In the supplementary sheet, the figure numbers don't follow linear order for better readability with main manuscript.**

Supplementary Note1: Plausible explanation for better performance of proposed DEXAT model

We use the pseudo-derivative framework Supplementary Equations (1)-(3) [1] to analyze the behaviour of our proposed DEXAT neuron in any generic RSNN. Supplementary Equation (1) denotes the pseudo-gradient of the neuron output spike. Supplementary Equation (2) represents the normalized membrane potential. Supplementary Equation (3) represents the error gradient of the hidden layer.

$$dz_j(t) / dv_j(t) = \gamma\, max\{0,\ 1 - |v_j(t)|\} \qquad \ldots\ldots\ldots\ldots(1)$$

$$v_j(t) = (V_m(t) - B_j(t)) / B_j(t) \qquad \ldots\ldots\ldots\ldots(2)$$

$$\Delta E = dE / dv_j(t) = dE / dz_j(t) * dz_j / dv_j(t) \qquad \ldots\ldots\ldots\ (3)$$

Here, z_j (t) denotes output spike of neuron 'j', γ denotes the damping factor having a value less than 1, V_m (t) denotes the neuron membrane potential and B_j (t) is the neuron firing threshold voltage, $dE/ dz_j(t)$ is the error derivative of the output layer. From Supplementary Equations (1)-(2), we observe that the pseudo-derivative is a function of the neuron's firing threshold B_j (t). Thus, the adaptive behaviour of the neuron threshold determines the magnitude and variation of the pseudo-derivative. Further, the hidden layer error gradient ΔESupplementary Equation (3) depends on the product of pseudo-derivative and error calculated at the output layer. Synaptic weight changes during training are governed by Supplementary Equation (4) and are function of hidden layer error gradient ΔE. Weight updates are generally given by Supplementary Equation (4). We have used ADAM optimizer equations for optimizing synaptic weight updates in our network.

$$w_t = w_{t-1} - \eta \Delta E \ldots\ldots\ldots\ldots\ldots\ (4)$$

Thus, synaptic weight changes are governed by the adaptive threshold decay behaviour. We frame a hypothesis based on above set of pseudo-derivative, error gradient and weight update Supplementary Equations (1)-(4)and some assertions to make a logical case below:

Assertion 1: Certain applications require large working memory

Sequential tasks (such as speech recognition), operating at real-time, have a large working memory requirement i.e need of several time steps for temporal processing (in revised manuscript we show 100 time steps of speech recognition, 280 time steps for SMNIST etc.). Thus to provide a long-short term memory to the network, large values of neuron adaptation time constants are necessary. Thus, in case of pure ALIF neurons, adaptation time constants equal-to or greater than the application's working memory are used for realizing the LSNN [1]. However, upon a deeper mathematical analysis (using pseudo-derivative and error gradient framework) we found that just using a single large adaptation time constant doesn't lead to the most optimized solution and more than one time constant of specific types can lead to better outcome.

Assertion 2: A smaller time constant assists the system in finding global minima and weight fine-tuning

From Supplementary Equation (3) we observe that a small pseudo-derivative value results in a small value of ΔE. Also, from Supplementary Equation (4) synaptic weight changes are governed by the magnitude of ΔE. A smaller time constant present in the adaptive threshold decay profile of the neuron in a LSNN can help achieve a small pseudo-derivative value (see Supplementary note 2 for mathematical details). Thus, only with the inclusion of a small time constant in decay profile, precise fine-tuning of weight-matrix can be performed during training to achieve global error minima and thus high accuracy. Hence, we need two time constants for the neurons constituting the LSNN. Larger time constant to satisfy the working memory requirement for sequential tasks while smaller time constant to enable subtle and precise weight matrix fine-tuning.

Assertion 3: Desired Activation Function behavior: (i) During training initially a smaller pseudo-derivative value should occur for a finite duration followed by a larger pseudo derivative value. (ii) Active neurons should experience lower value of pseudo-derivative while inactive neurons should experience higher value of pseudo-derivative for network fine-tuning and convergence.

During training, as specific neurons learn (or become sensitive to) certain specific features, their firing activity increases whenever they encounter similar features in the input stimuli. In order to consolidate learning, one would want to only minutely fine-tune (induce only small changes) the weight matrices of those neurons which have a higher firing activity (i.e. the ones which have already learnt certain feature(s)). Any drastic variation in the weight matrices of such neurons (which have already learnt something) may lead to loss of learning or even complete forgetting of the feature. From Assertion2, one can deduce that smaller pseudo-derivative value leads to finer weight changes and vice-versa. The proposed DEXAT model ensures that when a neuron fires frequently (i.e. smaller inter-spike interval) it automatically experiences a lower pseudo-derivative value (and consequently minute weight changes), due to our specific choice of a smaller first time constant in the DEXAT decay profile. Thus, having a faster time constant as the first time constant in DEXAT decay profile, reinforces the learning of the neuron. However, if the proposed DEXAT neuron fires infrequently (i.e. low activity, longer inter-spike time), it will encounter the second longer time constant from the decay profile (consequently inducing larger changes in its weight matrix). Further, since all the active neurons in the LSNN undergo fine tuning, a significant number of weights in the network auto converge independent of the decay of learning rate 'η' (after every 'n' iterations) in Supplementary Equation (4), that is conventionally done to accelerate convergence. Hence, the inherent behaviour of our proposed DEXAT neuron helps in achieving faster convergence of the network. Thus in the proposed DEXAT neuron model the decay profile is constructed with 2 time constants such that the smaller time constant occurs first followed by the larger time constant.

Supplementary Note 2: Mathematical analysis of performance improvement due to DEXAT

From Supplementary Equation (1) and (2) we can write pseudo-derivative as

$$\frac{dz_j(t)}{dv_j(t)} = \gamma\, max\left\{0,\ 1 - \left|\frac{V_m(t) - B_j(t)}{B_j(t)}\right|\right\} \text{-------------} \quad (5)$$

Two cases are possible w.r.t. the spike event

Case 1 : *(Spike event)* $V_m(t) \geq B_j(t)$

When membrane potential is greater than threshold voltage pseudo-derivative can be simplified to Supplementary Equation (6)

$$\frac{dz_j(t)}{dv_j(t)} = \gamma\, max\left\{0,\ \frac{2 * B_j(t) - V_m(t)}{B_j(t)}\right\} \text{----------} \quad (6)$$

Case 2 : $(After\ Spike\ event)\ V_m(t) < B_j(t)$

When membrane potential is less than threshold voltage pseudo-derivative can be simplified to Supplementary Equation (7)

$$\frac{dz_j(t)}{dv_j(t)} = \gamma\, max\left\{0,\ \frac{V_m(t)}{B_j(t)}\right\} \text{----------} \quad (8)$$

We analyze case 2 to see the effect of adaptation time constants and threshold decay on pseudo-derivative behaviour. After the spike event has occurred, the pseudo-derivative magnitude is governed by the ratio of membrane voltage and threshold voltage decay i.e. $\frac{dz_j(t)}{dv_j(t)} = \frac{V_m(t)}{B_j(t)}$(assuming γ=1). Hence, larger is the value of threshold voltage $B_j(t)$, smaller is the pseudo-derivative magnitude $\frac{dz_j(t)}{dv_j(t)}$ at a time 't'. In our proposed DEXAT model there are two time constants governing the threshold decay and the threshold voltage is given by Supplementary Equation (12).

$$B_j(t) = b_{j0} + \beta_1 b_{j1}(t) + \beta_2 b_{j2}(t) \quad \text{---------} \ (9)$$

$$b_{j1}(t + \delta t) = \rho_{j1} b_{j1}(t) + (1 - \rho_{j1}) z_j(t) \quad \text{--------} \ (10)$$

$$b_{j2}(t + \delta t) = \rho_{j2} b_{j2}(t) + (1 - \rho_{j2}) z_j(t) \quad \text{--------} \ (11)$$

where $b_{j1}(t)$ is the term corresponding to smaller time constant as in Supplementary Equation (10) and $b_{j2}(t)$ is the term corresponding to larger time constant in Supplementary Equation (11). Assuming $\tau_{a1} < \tau_{a2}$, during the initial time after spike $\sim(t < 5\tau_{a1})$ threshold decay is dominated by τ_{a1} but the total threshold voltage is governed by the sum of both the decay terms i.e. $b_{j1}(t)$ and $b_{j2}(t)$ according to Supplementary Equation (12). This results in a larger magnitude of threshold voltage $B_j(t)$. Hence, from Supplementary Equation (11) the magnitude of pseudo-derivative $\frac{dz_j(t)}{dv_j(t)}$ for initial duration ($\sim 5\tau_{a1}$) after spiking becomes small in DEXAT neuron. After the smaller time constant term $b_{j1}(t)$ decays completely for $\sim (t > 5\tau a_1)$ and reduces to almost zero, threshold voltage value magnitude is determined by a single larger time constant term $b_{j2}(t)$. Hence,$B_j(t)$ reduces thereby increasing pseudo-derivative $\frac{dz_j(t)}{dv_j(t)}$ gradually for later part of decay. This can be observed in Supplementary Fig. 2 for DEXAT neuron with two time constants τ_{a1}= 3 δt and τ_{a2}= 280 δt where δt denotes smallest time step. From Supplementary Fig.2 (c), (d) we observe that a small pseudo derivative value can be realized for a finite duration after firing event using a single small value of time constant (here 3 δt) in an ALIF neuron. However, in this case the threshold voltage decays rapidly and quickly reaches the baseline voltage. Hence, it cannot provide a practical working memory for temporal processing and defeats the motivation of using an adaptive neuron in RSNN for sequential tasks. On the other hand, a single large adaptation time constant (here 280 δt) satisfies the working memory requirement of

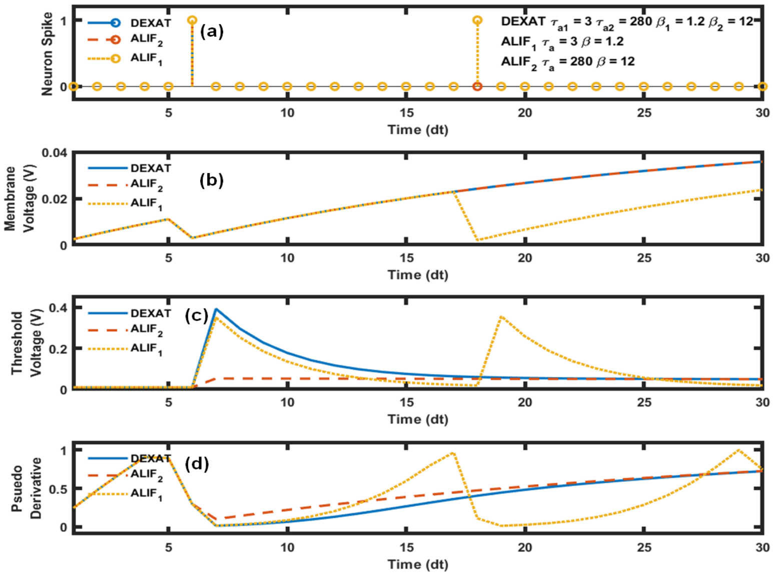

Supplementary Figure 2. Pseudo-derivative behaviour comparison for DEXAT and ALIF neurons. A constant current of 50 mA is injected as input to all the three neurons, $ALIF_1$ (with a small time constant) and $ALIF_2$ (with a large time constant) and a DEXAT neuron (with a small and a large time constant). Neuron parameters are listed inside the graph. (a) Output spikes corresponding to input current (b) Membrane potential evolution (c) Adaptive threshold behaviour and (d) Pseudo-derivative magnitude behaviour for the three neurons.

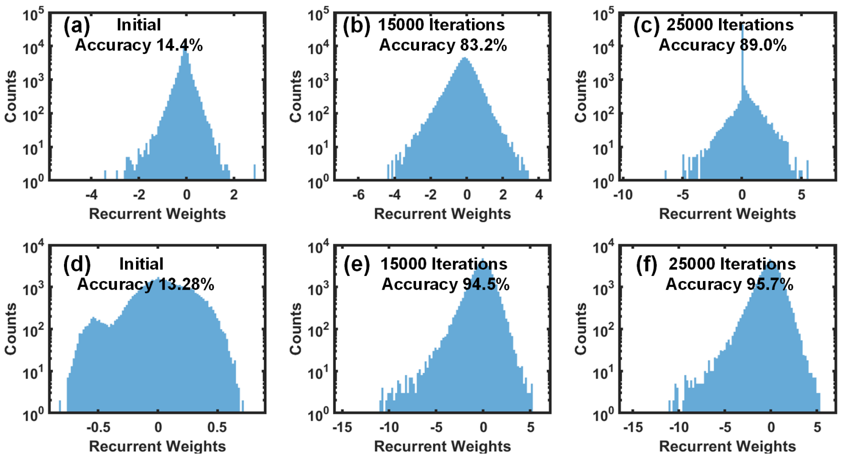

Supplementary Figure 3. Comparison of synaptic recurrent weight matrix evolution between ALIF and DEXAT based LSNN for SMNIST application. Recurrent weight distribution for ALIF based LSNN during training: (a) Initial random, (b) After 15000 iterations, and (c) after 25000 iterations. Synaptic recurrent weight distribution in DEXAT based LSNN during training: (d) Initial random, (e) After 15000 iterations, and (f) after 25000 iterations.

sequential tasks but is not able to provide the necessary small pseudo-derivative magnitude for some duration after firing event compared to DEXAT neuron as shown in Supplementary Fig. 2 (d). We observe from Supplementary Fig. 2 (c) that threshold voltage in our DEXAT neuron achieves a high value due to the presence of two contributing time constant terms as given in Supplementary Equation (7). This results in a smaller pseudo-derivative value (from Supplementary Equation (6)) for some duration just after spike when compared to ALIF neuron as shown in Supplementary Fig. 2 (d). From Supplementary Fig. 2 (c) we note that a small initial time constant also ensures that the larger threshold voltage achieved after spike decreases for some time so that pseudo derivative doesn't stay at a low value for a large duration else there are chances of the network getting stuck in local minima. Hence, DEXAT neuron helps in achieving high accuracy and faster convergence while satisfying large working memories.

We analyze a case of training an ALIF based- versus a DEXAT based- LSNN. For this we extracted the synaptic weights during the training procedure for SMNIST using the 3 layer LSNN network as shown in Fig. 1 (c) of main manuscript. The network consists of 80 input neurons, 120 LIF and 100 DEXAT neurons in the hidden layer and 10 output neurons. We analyzed synaptic weight distribution at the start of training (recurrent weights shown), after 15000 iterations and after 25000 iterations for both the networks as shown in Supplementary Fig. 3. It can be observed in Supplementary Fig. 3 (e)-(f) that in the case of DEXAT based LSNN weight distribution do not drift much while going from 15000 training iterations to 25000 training iterations, which can be considered as fine-tuning of weight matrices (i.e. no drastic changes as accuracy improved from 94.5% to 95.7%). This happens due to the advantage of two time constants as described earlier. However, in case of ALIF based LSNN there is a significant drift in weight distribution while going from 15000 training iterations to 25000 iterations as evident in Supplementary Fig. 3 (b)-(c) , indicating that weight-tuning was coarse and more drastic (as accuracy improved from 83.2% to 89.1%).

Supplementary Note 3: Digital implementation of DEXAT neuron block

For a fully digital implementation of our DEXAT threshold modulator function using a FPGA, approximation of exponential terms governing adaptive threshold decay in Supplementary Equation (10) and (11) becomes a necessity. Expansion of the exponential term is given by Supplementary Equation (12).

$$\exp(-\delta t/\tau_a) = 1 - \delta t/\tau_a + \delta t/\tau_a^2 + \delta t/\tau_a^3 + \ldots\ldots (12)$$

Also, based on the required precision, the number of terms to be included in the expansion of (12) can be decided while implementing the digital DEXAT block. For a 16 bit fixed point precision exponential function in Supplementary Equation (10)-(11) can be approximated to three terms while for a reduced 8 bit fixed point

precision, only two terms of exponential expansion are needed in Supplementary Equation (10)-(11). As shown in Supplementary Table 1, increased precision comes at a large hardware implementation cost. Supplementary Fig. 8 shows the DEXAT adaptive behaviour simulated and synthesized for FPGA implementation in Verilog for 16 bit fixed point precision.

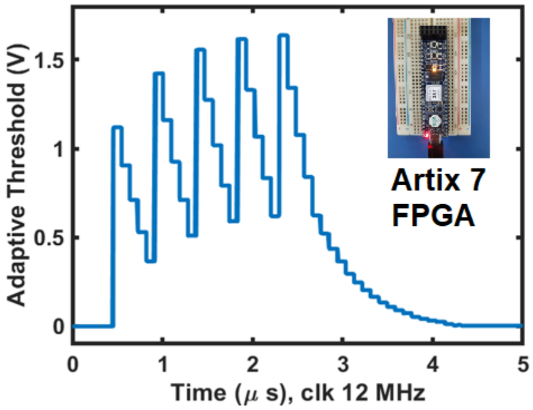

Supplementary Figure 8. Digital implementation of DEXAT neuron behaviour. DEXAT adaptive threshold behaviour obtained using 16 bit precision on Xilinx Artix 7 FPGA.

Supplementary Table 1: FPGA implementation of DEXAT neuron block.

Number system	Total Bits	Decimal Bits	Tau Limit (dt)	LUTs	Reg	DSP
**Fixed Point**	8	4	32	79	41	0
**Fixed Point**	16	8	512	22	33	6
**Floating Point**	32	IEEE754	No limit	3091	1361	12

Supplementary note 4: Performance estimation of proposed DEXAT neurons

We estimate the energy consumed by our DEXAT neuron used for threshold modulation in a LSNN network per inference for different application tasks. For this, we extract SET/RESET current flowing in our DEXAT circuit for our sample sequence S1. We empirically correlate the increment in threshold voltage (Δ) at each spike event with corresponding increase in average current flowing in circuit with consecutive applied SET pulses. We observe that the magnitude increment in threshold voltage at each spike event is directly proportional to the magnitude increment in average SET current for consecutive spikes (see programming pulse strategy discussed in main manuscript). Hence, extracting SET and RESET current behaviour for our programming scheme energy estimate can be calculated for an arbitrary firing activity of DEXAT neuron.

Supplementary Table 2 shows estimated "energy/per-inference" for threshold-modulation block of the proposed DEXAT neuron for (i) Speech recognition and (ii) SMNIST classification applications. For SMNIST classification using DEXAT based LSNN (100 DEXAT neurons) 60000 images were used for full dataset inference. Estimated inference energy dissipation/per DEXAT for each of the 60000 inferences are shown in Supplementary Fig. 12. It can be observed that energy values are scattered over a range. This can be attributed to the instantaneous firing patterns and activity of the hidden layer neurons. To account for the activity dependent energy dissipation nature of DEXAT neurons, it is essential that energy be reported in context of specific applications. Thus, in Supplementary Table 2 we report maximum, median and minimum energy per inference for SMNIST and speech recognition applications for the DEXAT LSNNs shown in the manuscript. In Supplementary Table 2, we compare with literature reported dissipation values for other relevant adaptive

Supplementary Table 2: Estimated energy benchmarking with other relevant adaptive neurons.

Ref.	Neuron Type and Technology	Energy/Power	Remarks	Method
[2]	-Mihalas Neibur model, Single exponential<br>-0.5 um CMOS based Adaptive circuit	40 nW* @ 5V	-no network application shown<br>- fabricated	Simulated energy values per-spike (Reported in literature)
[3]	ADeX Model, Single exponential<br>65 nm CMOS based Adaptive circuit	Energy: 200 pJ$ @ 300Hz<br>Avg.power: 60nW$	- no network application shown<br>- 32 neuron array fabricated	
[4]	-RRAM based Adaptive circuit,<br>-Single exponential<br>65 nm CMOS + RRAM	Not reported	-simulated adaptive neuron circuit<br>-MNIST using SNN	Not reported
**This Work**	- DEXAT Model, Double exponential<br>-Ni/HfO2/Al doped TiO2/TiN OxRAM<br>-50 um cross point OxRAM +<br>-0.5 um CMOS circuit<br>-Energy reported for threshold modulation block	1.75 mJ (Max)<br>298 µJ (Median)<br>10µJ (Min)	Energy per inference for SMNIST classification	Measured
		696 µJ (Max)<br>116 µJ (Median)<br>13 µJ (Min)	Energy per inference for Speech recognition	
	-DEXAT Model, Double exponential<br>-Mo/TiOx/TiN device [500 nm cross point RRAM]<br>-Energy reported for threshold modulation block	58 µJ (Max)<br>11.12 µJ(Median)<br>1.85 µJ (Min)	Energy per inference for SMNIST classification	Projected using values from [5]
		22.27 µJ (Max)<br>3.57 µJ (Median)<br>0.24 µJ (Min)	Energy per inference for Speech recognition	
	-DEXAT Model, Double exponential<br>-Mo/TiOx/TiN device [30 nm cross point RRAM]<br>-Energy reported for threshold modulation block	0.77 µJ(Max)<br>0.13 µJ (Median)<br>3.38 µJ (Min)	Energy per inference for SMNIST classification	
		0.3 µJ (Max)<br>46.2 nJ (Median)<br>0.4 nJ (Min)	Energy per inference for Speech recognition	

* Power is reported only for membrane and threshold modulation blocks excluding comparator and other peripheral blocks.

$ Energy/power is reported for full neuron circuit.

neuron circuits [2]-[4]. The Ni/HfO_2/Al doped TiO_2/TiN based circuit, characterized in this work, has an OxRAM device crosspoint dimension of 50 µm and CMOS selector of 5 µm length (@ 5V VDD), thus its energy consumption is in the order of ~ microjoules. Further, in Supplementary Table 2 we estimate projected dissipation of DEXAT threshold modulator block when scaled OxRAM devices are used. A 500 nm OxRAM device [5] results in ~ 27 X reduction, while a 30 nm device [5] results in ~2292 X reduction, in energy respectively, compared to the 50 µm device. Although we report the power/energy only of the threshold

modulator block (as other blocks are implemented in software), total energy/power of a neuron circuit built using proposed DEXAT adaptation block would be of the same order as that of individual DEXAT block and hence the comparison is valid.

Further, we perform a detailed performance (power, energy, area) benchmarking across multiple simulated CMOS technology nodes (180 nm, 90 nm, 65 nm, 28 nm) and FPGA for digital implementation of our proposed DEXAT neuron (Supplementary Table 3). Description of DEXAT neuron threshold modulator block is first defined in Verilog HDL. Next, digital ASIC DEXAT threshold modulator block is synthesized and simulated using multiple CMOS technology node libraries in Cadence Encounter tool to estimate approximate power dissipation and area. It is important to note that the digital blocks are as-synthesized and not additionally optimized or handcrafted. We observe a clear power and area scaling trend with the technology node till 28 nm. The verilog implementation is also synthesized on a Xilinx Artix 7 FPGA board using Xilinx Vivado tool to estimate power consumption for different precision. As expected, power and area increase with precision due to increased resource utilization on the FPGA. Energy consumption of digital threshold modulator block for SMNIST and speech recognition tasks was found to be in the order of ~ nanojoules and even ~ picojoules for some cases. From Supplementary Table 3, area overhead for digital implementations was found to be much larger compared to equivalent OxRAM based circuits.

Supplementary Table 3: Performance estimates of digital DEXAT implementation modulator block.

**Type of Digital Implementation & foundry**	**Precision**	**Power**	**Avg. Energy/per Inference -** SMNIST Classification (J)	**Avg. Energy/per Inference -** Speech Recognition (J)	**Area** (μm^2)
ASIC @ SCL180 nm (simulated)	16 bit fixed point	1.24 mW	8.68E-08	1.14E-08	82658.5
	Floating point	12.45mW	8.72E-07	1.14E-07	675717.9
ASIC @ UMC 90nm (simulated)	16 bit fixed point	0.11 mW	7.70E-09	1.01E-09	32586
	Floating point	2.10 mW	1.47E-07	1.93E-08	397007
ASIC @ TSMC 65nm (simulated)	16 bit fixed point	45.19 µW	3.16E-09	4.14E-10	11435.7
	Floating point	1.24 mW	8.68E-08	1.14E-08	88377.1
FPGA (Xilinx Artix 7 xc7a35tcpg236) (28 nm)	16 bit fixed point	453 µW	3.17E-08	4.15E-09	-
	Floating point	4.63 mW	3.24E-07	4.24E-08	-

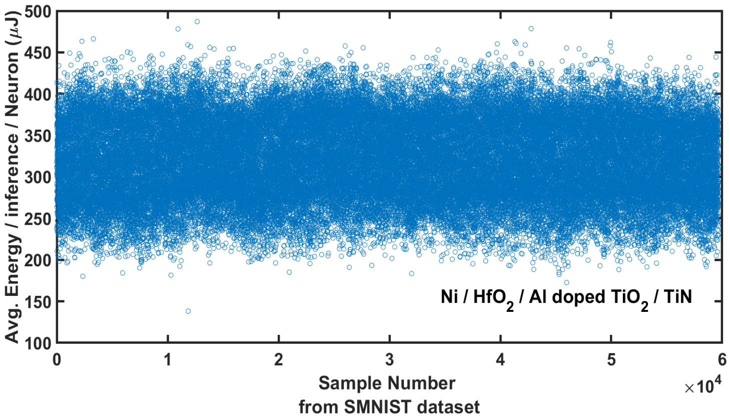

Supplementary Figure 12. Energy consumption per inference task for SMNIST task. 60000 sample images are used for inference. Average DEXAT threshold modulation energy per neuron in a single inference is calculated and plotted for each inference task involving 100 DEXAT neurons in the hidden layer of LSNN.

Supplementary note 5: OxRAM device endurance analysis

We estimated the statistics (min, max, mean) of programming cycles (SET/RESET) that an OxRAM device inside a hidden layer DEXAT neuron would undergo during inference for all 3 datasets presented in the paper (SMNIST, speech recognition, store-recall). These cycling statistics are shown in Supplementary Table 4. As per our analysis, if RRAM devices have endurance < 10^6 cycles then it is preferable to train the network offline using the proposed DEXAT model and use RRAM based low-power deployment on full hardware for inference tasks. Depending upon the device endurance, some minor in-situ training may still be possible on hardware. Training can also be done using digital implementations such as state of the art FPGAs as they will not pose an endurance issue.

Use of RRAM for in-situ training cannot be completely ruled out as there are multiple studies in literature indicating high RRAM endurance of 10^{12} cycles in [6], [7] for bi-layer stacks similar to the one used in our work. Supplementary Table 4 shows the impact of network size on device cycling statistics. It was observed that as network size increases mean cycling events per device decrease. Hence, the endurance requirement per device in the hidden layer tends to relax. Thus, larger hidden layers can help to bring down individual device stress for specific applications. Further, based on device cycling statistics we estimate the number of inferences (for each application) that a full hardware can perform before reaching the endurance limit. This analysis was done assuming networks built from RRAM devices of three different types, spanning a endurance range of 6 order of magnitude (Device 1: 10^6 cycles , Device 2: 10^9 cycles, and Device 3: 10^{12} cycles).

Further, we also model the impact of device resistance window degradation with cycling on network performance. As RRAM devices undergo cycling, the resistance window tends to squeeze. Authors in [8] studied the effect of endurance degradation in analog RRAM devices. Using this study as the basis [8] we modeled R_{on} / R_{off} ratio degradation to carry out dynamic LSNN inference simulations where DEXAT neuron behavior changes actively with the number of cycles. Modeled degradation of DEXAT neuron threshold voltage curves with cycling is shown in Supplementary Fig. 13 and Supplementary Fig. 14. Next, we inject these new degraded DEXAT curves dynamically in the network based on inference cycle/neuron activity count and re-estimate the network accuracy. Supplementary Table 5 shows the impact of resistance window degradation on network inference accuracy. We observe that the network is able to maintain a sufficiently high accuracy even for ~ 44% drop in the original R_{on} / R_{off} ratio.

Supplementary Table 4: Estimation of programming cycle requirement/ per DEXAT neuron depending on network size and application. Table also shows the approximate number of inferences before device breakdown.

**Application** (Dataset)	**Network Size** (LIF-DEXAT)	**Cycling event statistics/per inference** (for hidden layer DEXAT neurons)			**No. of inferences that can be supported*** (with different ideal device endurance)		
		**Max**	**Min**	**Mean**	**10^6 cycles**	**10^9 cycles**	**10^{12} cycles**
**SMNIST** (Statistics on 10,000 test dataset inferences)	12-10	99	0	60	~ 10.1×10^3	~10.1×10^6	~10.1×10^9
	30-25	87	0	31	~ 11.5×10^3	~11.5×10^6	~11.5×10^9
	120-100	61	0	20	~ 16.4×10^3	~16.4×10^6	~16.4×10^9
**Speech (2 class)** (Statistics on 734 test dataset inferences)	10-10	58	0	34	~ 17.2×10^3	~17.2×10^6	~17.2×10^9
	50-50	11	0	2	~ 90.9×10^3	~90.9×10^6	~90.9×10^9
**Speech (12 class)** (Statistics on 4890 test dataset inferences)	100-100	486	0	86	~2.1×10^3	~2.1×10^6	~2.1×10^9
	300-300	434	0	79	~2.3×10^3	~2.3×10^6	~2.3×10^9
	500-500	445	0	52	~2.2×10^3	~2.2×10^6	~2.2×10^9
**STORE-RECALL**	10-10	39	0	16	~ 25.6×10^3	~25.6×10^6	~ 25.6×10^9

* Number of supported inferences are calculated assuming that each device undergoes the ‘max’ (i.e. worst case) number of cycling events in each inference.

Supplementary Table 5: Test accuracy degradation with cycling for SMNIST task (each run corresponds to inference on 10000 test images using LSNN with 120 LIF and 100 DEXAT neurons).

**Cycles** (number of programming hits taken by the device during inference)	**< 10^6 cycles**	**10^6-10^7 cycles**	**10^7-10^8 cycles**	**10^8-10^9 cycles**	**> 10^9 cycles**
**(a) *Drop in resistance window** (Ron/ Roff) %	0 %	11 %	44 %	54 %	74 %
**(i) Test Accuracy** (%) [with resultant variability = 10%]	95.4 %	94.6 %	87.5 %	77.9 %	43 %
**(ii) Test Accuracy** (%) [with resultant variability = 30%]	93.2 %	92.6%	80.9 %	70. %	37.6%
**(b) **Drop in resistance window** (Ron/ Roff) %	0 %	20 %	40 %	60%	80 %
**Test Accuracy** (%) [Resultant variability = 30%]	93.2 %	92.3%	88.5 %	62.5%	39.7%

*In this case RRAM resistance window degradation corresponds to data extracted from [8].
**In this case RRAM resistance window degradation is assumed to be uniform 20% in each window.

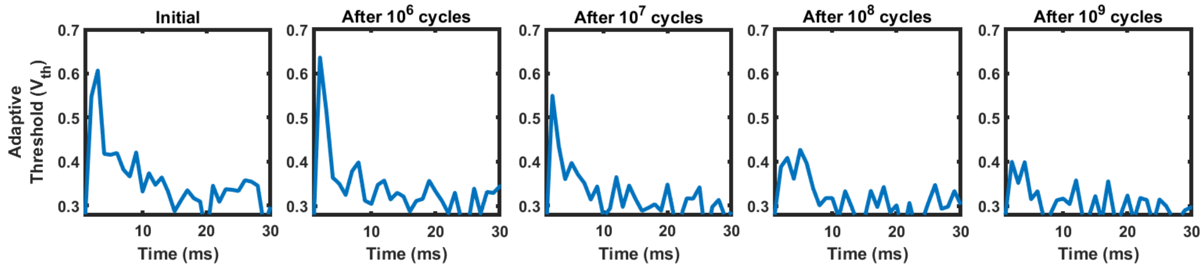

Supplementary Figure 13: Dynamic modeling of degradation of adaptive threshold decay behaviour. Device R_{on} / R_{off} ratio degrades with increasing number of programming cycles. Scaling of Ron/Roff ratio is based on characterization shown in [8]. Note overall window squeezes with cycling.

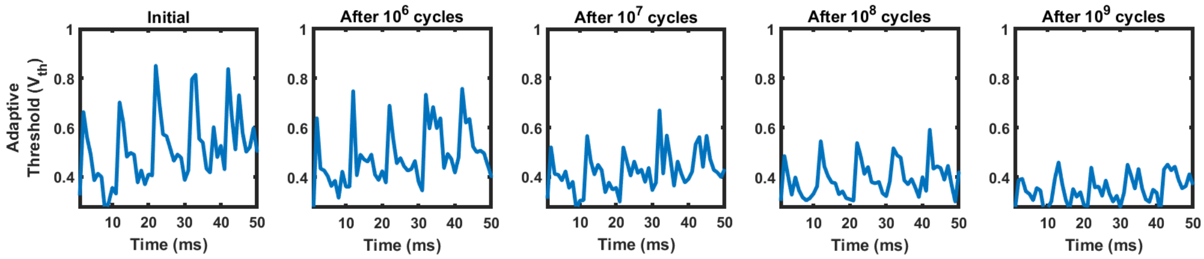

Supplementary Figure 14: Post cycling DEXAT behaviour for multiple spike events. Simulated DEXAT neuron behaviour for multiple spike events after injecting dynamic cycle-lifetime based threshold decay curves for respective hidden layer neurons in the LSNN simulations. After a large number of cycles almost no DEXAT action is observed.

Supplementary Figures and Tables

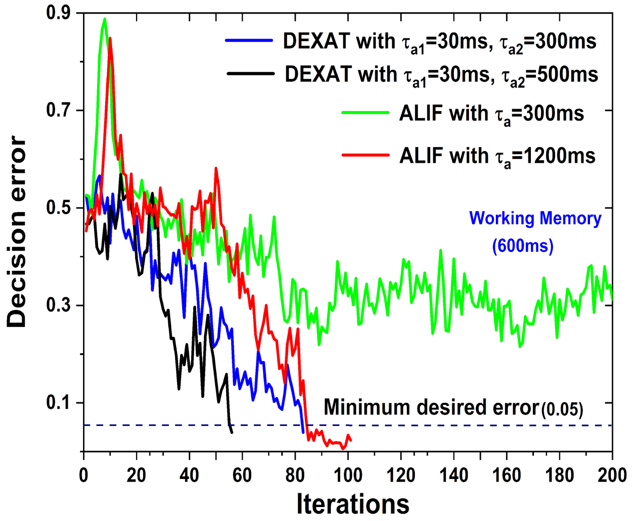

Supplementary Figure 1. STORE and RECALL task for working memory of 600 ms. Decision error on STORE and RECALL task using DEXAT and ALIF neurons with different time constants for a working memory requirement of 600 ms.

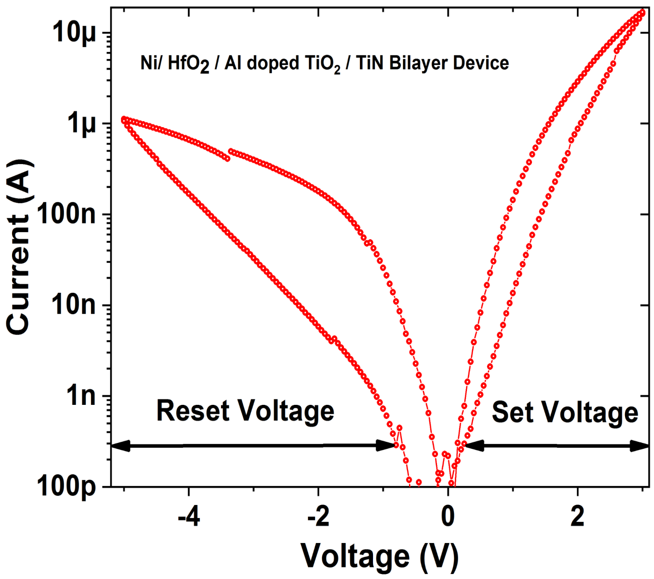

Supplementary Figure 4. DC I-V curve of bilayer non-filamentary device. DC Characterization of Ni/ HfO_2 /Al doped TiO_2/TiN device.

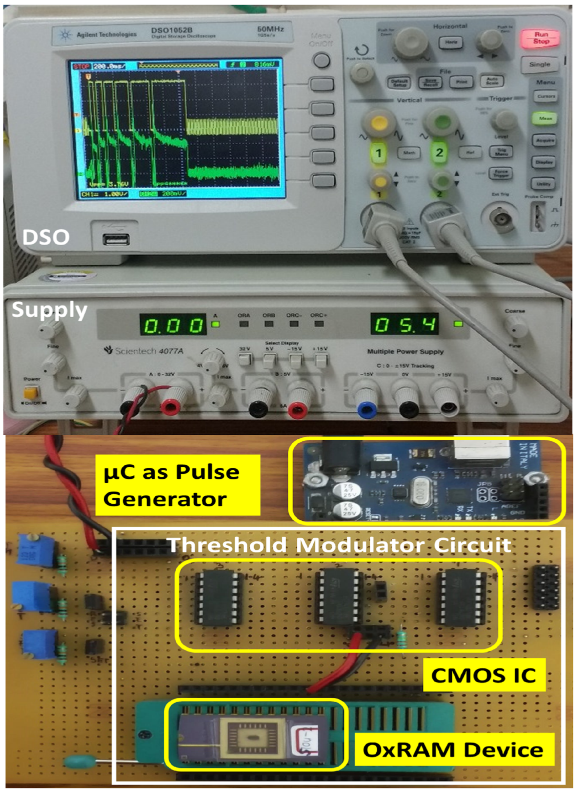

Supplementary Figure 5. Experimental setup used for sample sequences (i.e. *S1* and *S2*) testing to demonstrate DEXAT behaviour. Experimental setup showing neuron circuit on General purpose board, Digital Storage Oscilloscope (DSO) and external power supply.

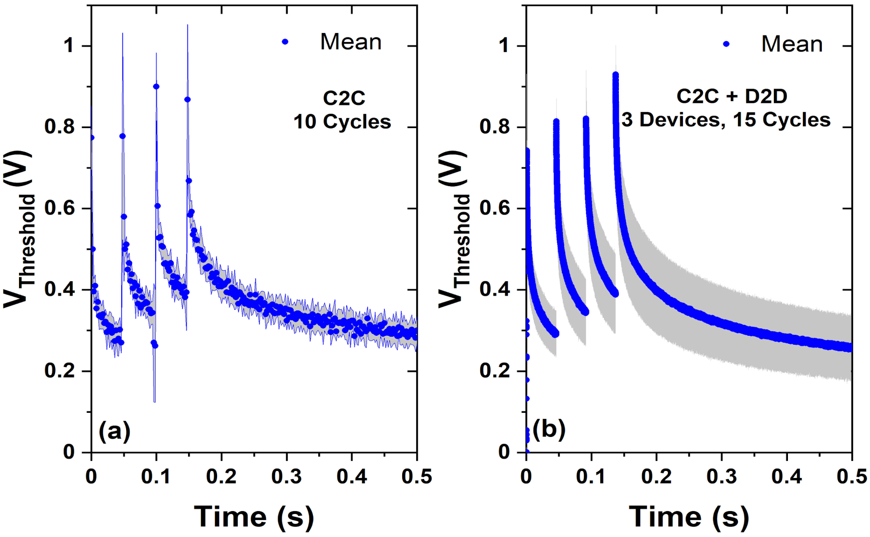

Supplementary Figure 6. Experimental variability measurements for adaptive threshold voltage for sample sequence S1 on Ni/HfO_2 /Al doped TiO_2/TiN device. (a) C2C variability, showing mean and standard deviation for each point. (b) C2C+D2D variability (obtained by cycling multiple devices multiple times), showing mean and standard deviation for each point. Solid blue dots represent the mean of each threshold voltage point and shaded grey region denotes the standard deviation. Variance for C2C+D2D case is higher than variance for C2C only case.

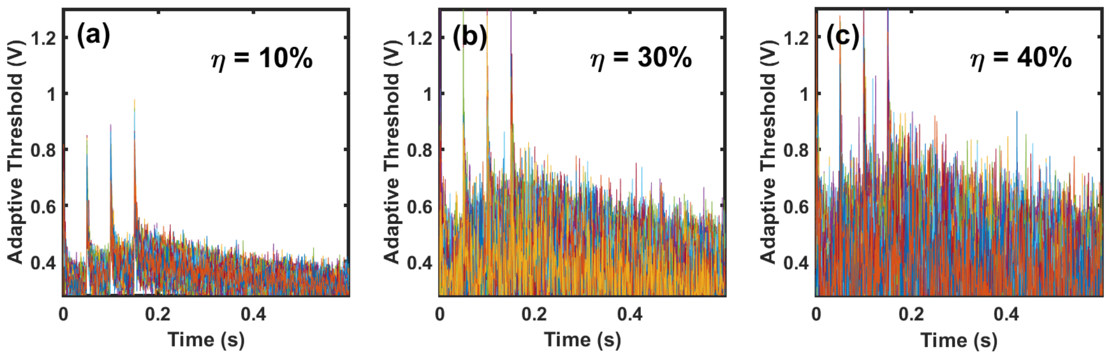

Supplementary Figure 7. Simulated DEXAT neuron adaptive threshold cycles using resultant variability parameter. Each curve shows 10,000 simulated neuron traces for sequence S1 capturing effect of both C2C+D2D variability. 10,000 traces are representative of 100 neurons for 100 cycles (or X neurons for 10,000 / X cycles). (a) η_r = 10 %, (b) η_r = 30 % and (c) η_r = 40 %.

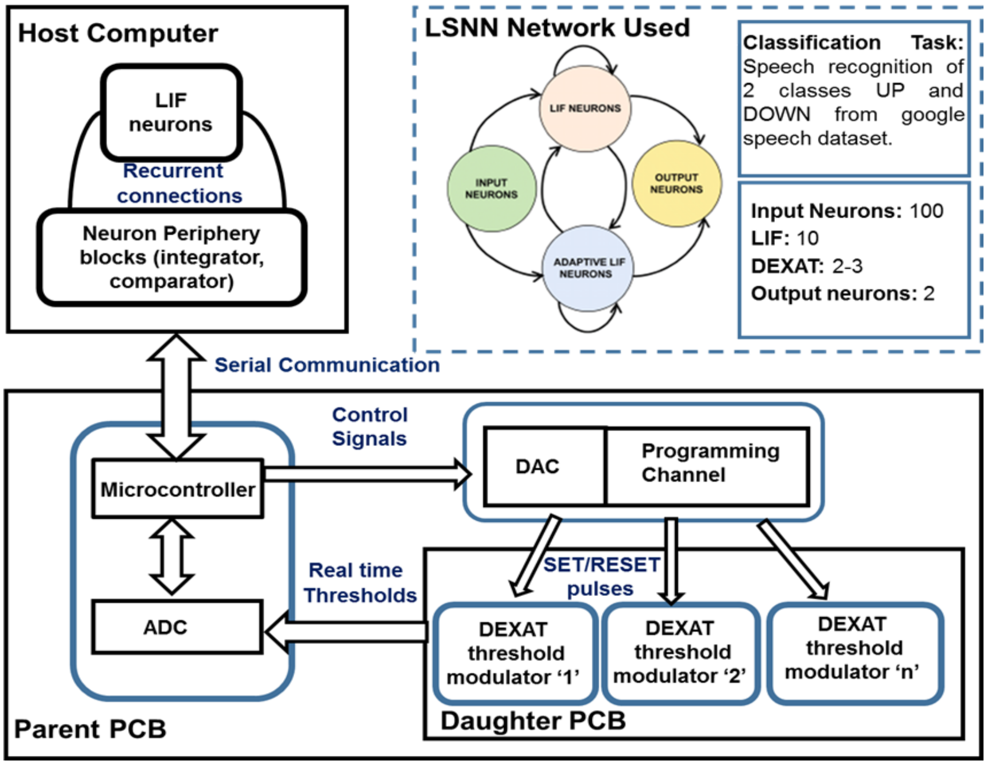

Supplementary Figure 9. Experimental setup for full end to end speech recognition task. Schematic of the designed experimental setup showing communication between different modules

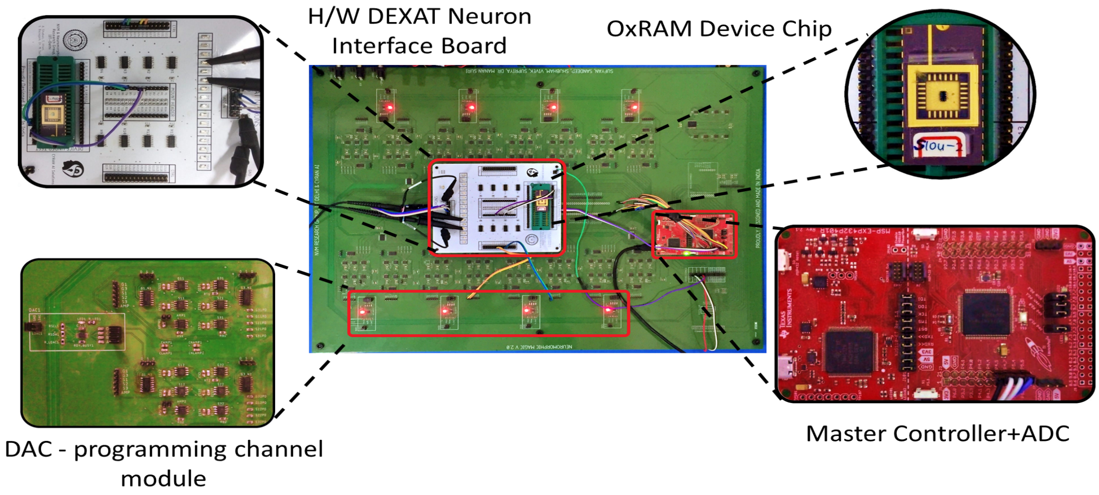

Supplementary Figure 10: Real time experimental setup showing fabricated PCB for end to end speech recognition task. Fabricated PCBs showing parent board with required blocks like ADC, microcontroller, DAC and interface board with DEXAT neurons.

(a) Speech sample with label 'DOWN'

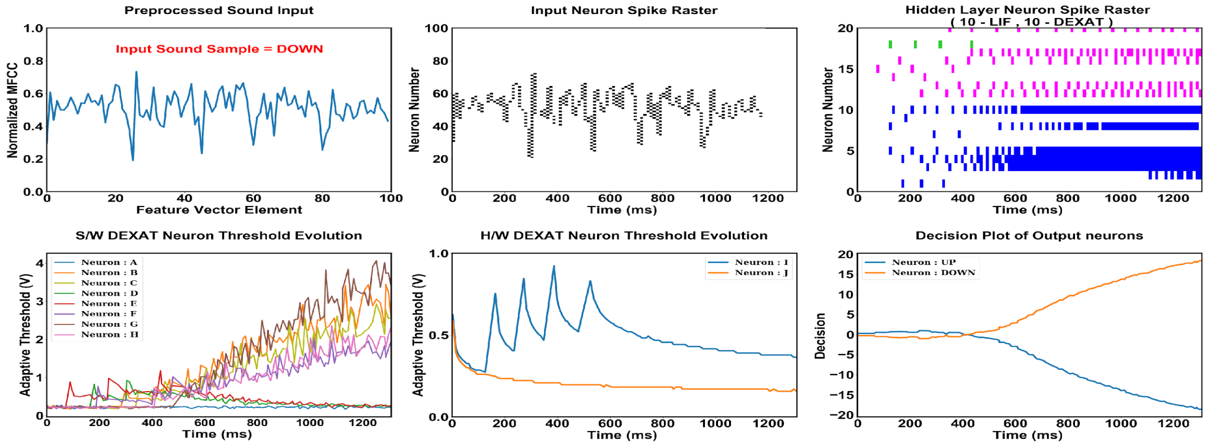

(b) Speech sample with label 'UP'

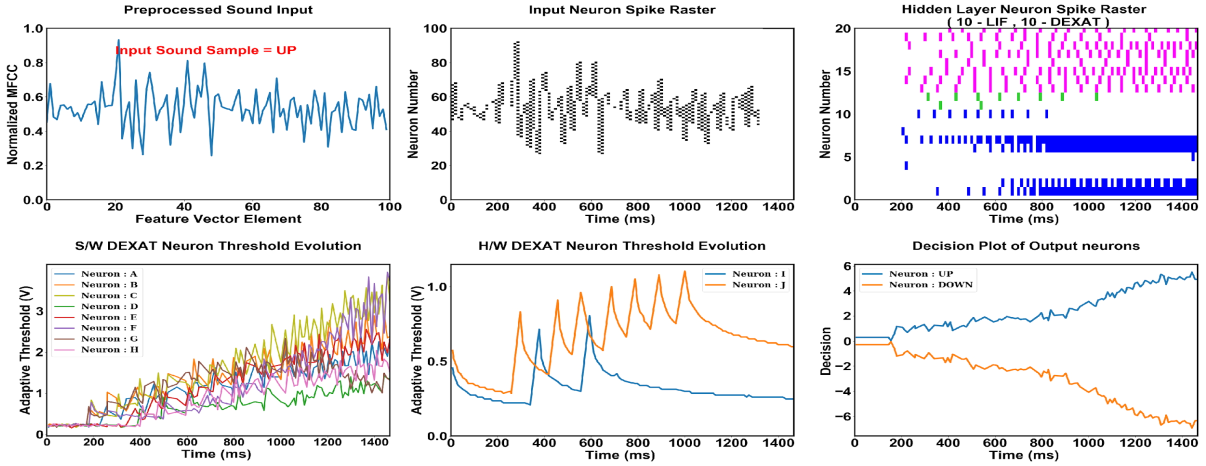

(c) Speech sample with label 'DOWN'

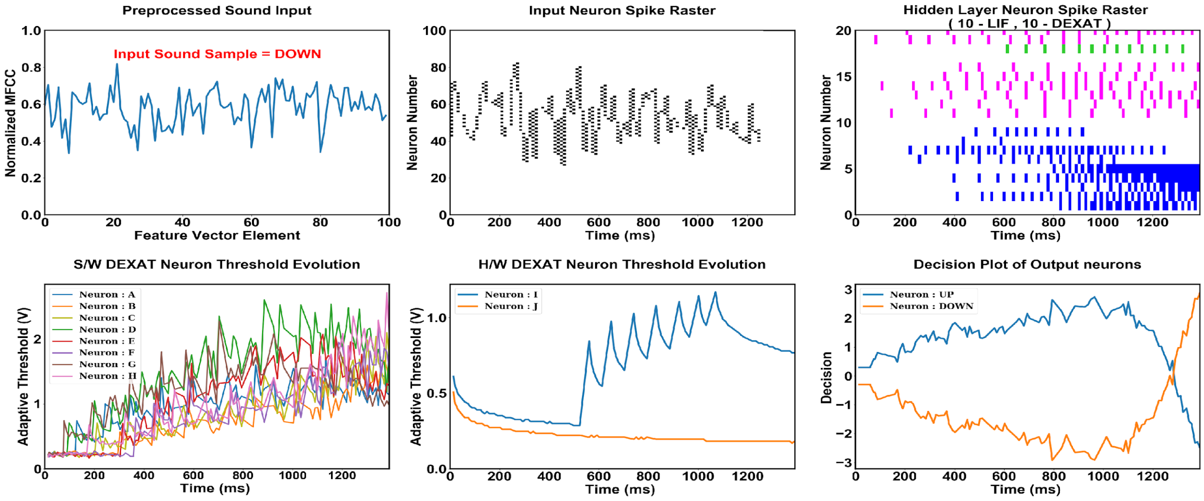

(d) Speech sample with label 'DOWN'

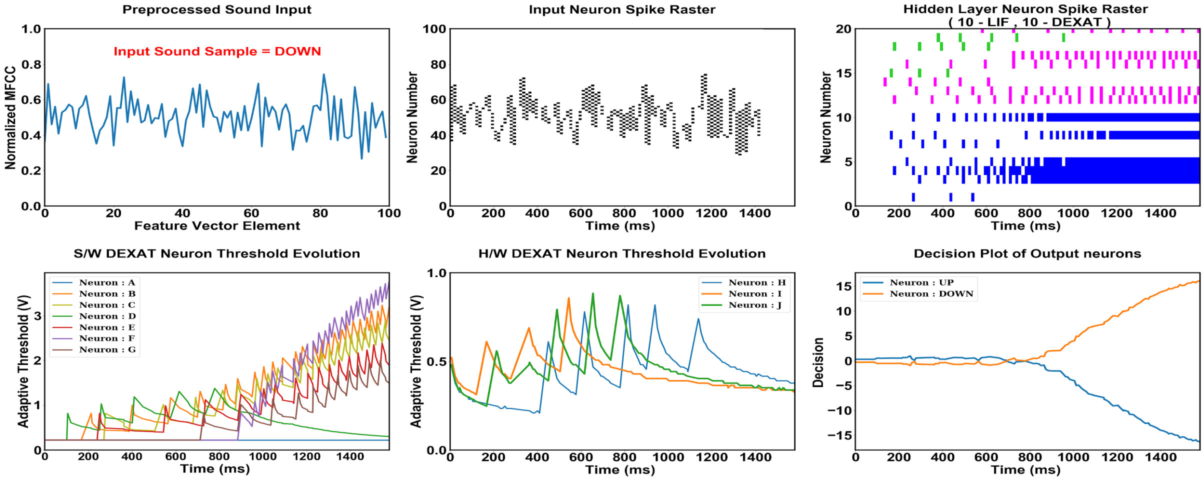

(e) Speech sample with label 'DOWN'

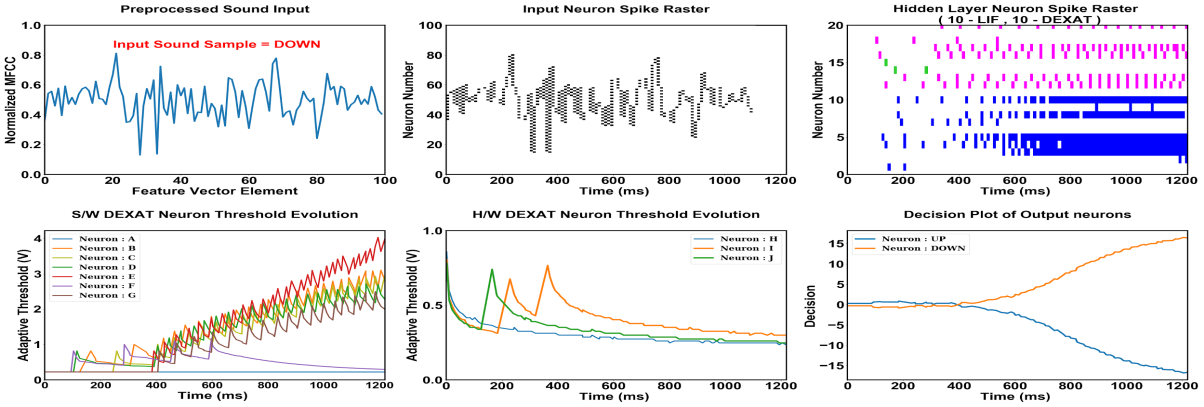

(f) Speech sample with label UP’

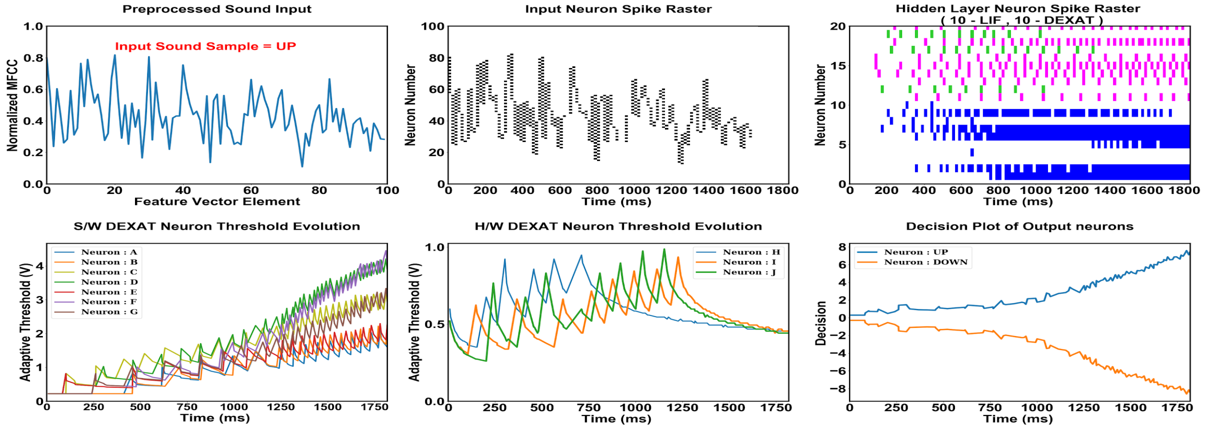

Supplementary Figure 11. Speech recognition task using hardware and software DEXAT LSNN. Speech recognition results for speech samples taken randomly from google speech dataset for two classes ‘UP and ‘DOWN’ using LSNN with (a)-(c) 2 H/W DEXAT neurons,(d)-(f) 3 H/W DEXAT neurons.

Supplementary Table 6: Network architecture details used in STORE-RECALL, SMNIST and end to end speech recognition task (2 Class GSC)

**Network Layer**	**SMNIST classification task Architecture**		**STORE-RECALL Architecture**		**End to End Speech Recognition task Architecture (2 Class GSC)**	
	**No. of neurons**	**Synaptic Weights**	**No. of neurons**	**Synaptic Weights**	**No. of neurons**	**Synaptic Weights**
**Input Layer**	80	(80 x 220) = 17,600	100	(100x20) = 2000	100	(100x20) = 2000
**Hidden layer**	120 LIF + 100 DEXAT	(220x220) = 48,400	10 LIF + 10 DEXAT	(20x20) = 400	10 LIF + 10 DEXAT	(20x20) = 400
**Output Layer**	10	(220x10) = 2,200	10	(20x10) = 200	2	(20x2) = 40

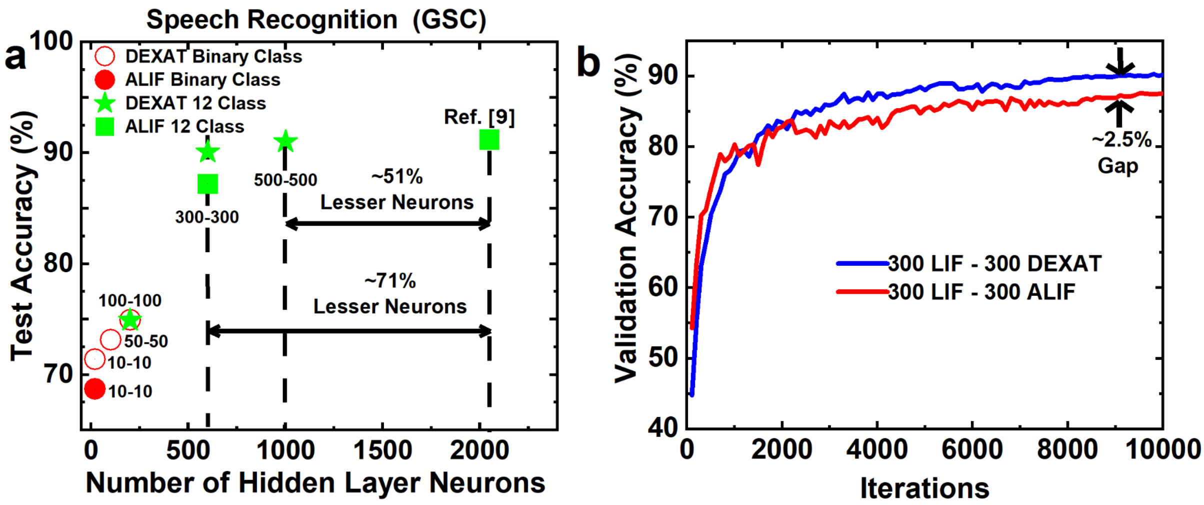

Supplementary Figure 15 LSNN classification performance on GSC dataset. (a) Test accuracy vs network dimension. DEXAT based LSNN achieves higher accuracy compared to ALIF based LSNN even for significantly fewer hidden layer neurons. (State-of-the-art-accuracy [9] is achieved even with ~51% lesser neurons). All binary class simulations are performed after training the LSNN on a reduced 2-class GSC dataset. DEXAT binary class result for network size (10-10) corresponds to the end-to-end speech experiment described in manuscript. Also, it can be seen that accuracy increases with increasing network size for both binary and 12-class GSC. **(b)** Test accuracy comparison for a 300-300 sized ALIF vs DEXAT LSNN. For all GSC simulations, DEXAT neurons are used based on the hardware extracted parameters on HfO_2 / TiO_2 device.

Supplementary References:

[1] Bellec, G., Salaj, D., Subramoney, A., Legenstein, R. & Maass, W. Long short-term memory and learning-to-learn in networks of spiking neurons. In Advances in Neural Information Processing Systems, 787–797 (2018)

[2] Folowosele, F., Hamilton, T. J. & Etienne-Cummings, R. Silicon modeling of the mihalas–niebur neuron. IEEE transactions on neural networks 22, 1915–1927 (2011). 436 23.

[3] Aamir, S. A. et al. A mixed-signal structured adex neuron for accelerated neuromorphic cores. IEEE transactions on biomedical circuits systems 12, 1027–1037 (2018). 438 24.

[4] Wang, X. et al. A novel rram-based adaptive-threshold lif neuron circuit for high recognition accuracy. In 2018 International Symposium on VLSI Technology, Systems and Application (VLSI-TSA), 1–2 (IEEE, 2018).

[5] Park, J. et al. Tio x-based rram synapse with 64-levels of conductance and symmetric conductance change by adopting a hybrid pulse scheme for neuromorphic computing. IEEE Electron Device Lett. 37, 1559–1562 (2016).

[6] Hsu et al. Self-rectifying bipolar TaO x/TiO 2 RRAM with superior endurance over 10^{12} cycles for 3D high-density storage-class memory. *2013 Symposium on VLSI Technology*. IEEE, 2013.

[7] Lee et al. A fast, high-endurance and scalable non-volatile memory device made from asymmetric Ta_2O_{5-x} / TaO_{2-x} bilayer structures. *Nature materials* 10.8 (2011): 625-630.

[8] Zhao et al. Characterizing endurance degradation of incremental switching in analog RRAM for neuromorphic systems. *2018 IEEE International Electron Devices Meeting (IEDM)*. IEEE, 2018.

[9] Salaj, D. et al. Spike-frequency adaptation provides a long short-term memory to networks of spiking neurons. bioRxiv (2020). https://www.biorxiv.org/content/early/2020/05/12/2020.05.11.081513.full.pdf.
