# Supplementary figures and images for: An adaptive threshold neuron for recurrent spiking neural networks with nanodevice hardware implementation
Source: Nat Commun. 2021 Jul 9;12:4234. doi: 10.1038/s41467-021-24427-8 (PMC8270926; doi:10.1038/s41467-021-24427-8)

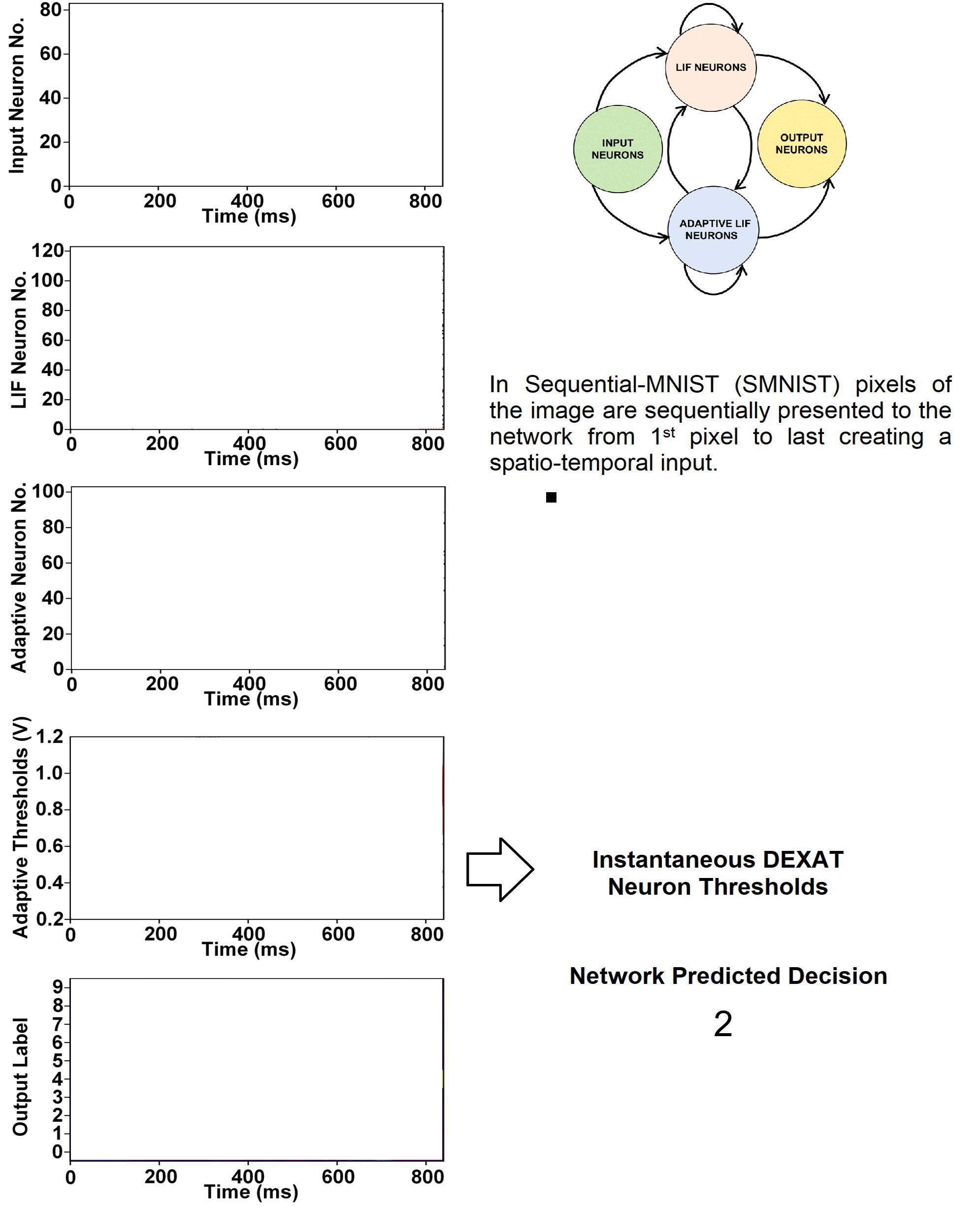

Supplement: Supplementary file 2 — Supplementary Movie 1 [file 41467_2021_24427_MOESM2_ESM.gif]
