# Supplementary material for: An adaptive threshold neuron for recurrent spiking neural networks with nanodevice hardware implementation
Source: Nat Commun. 2021 Jul 9;12:4234. doi: 10.1038/s41467-021-24427-8 (PMC8270926; doi:10.1038/s41467-021-24427-8)
Supplement: Supplementary file 4 — Description of Additional Supplementary Files [file 41467_2021_24427_MOESM4_ESM.pdf]

Description of Additional Supplementary Files

Title: Supplementary Movie 1

Description: Network dynamics and performance of an LSNN with DEXAT neurons on sequential MNIST task. In classifying Sequential-MNIST (SMNIST) task, pixels of the image are sequentially presented to the network after pixel encoding, which results in spiking activity of LIF and DEXAT neurons. Instantaneous adaptive threshold of eleven DEXAT neurons, which show the highest activity (i.e. whose mean threshold is greater than 1.5 times of maximum threshold) in the network is shown. Output neurons predict the class at each timestamp, which is shown as a network predicted decision

Title: Supplementary Movie 2

Description: We show real-time spoken speech recognition with our fabricated experimental setup consisting of multiple CMOS-OxRAM hardware DEXAT neurons working in unison with their software counterparts. A human subject speaks a speech command taken from Google speech dataset. The input real-time speech sample is pre-processed and given as input to our full end to end experimental setup realizing a DEXAT based LSNN. The network is partitioned into software and hardware blocks. Input speech command is recognized in real time by the network.
